# High glycemic albumin representing prestroke glycemic variability is associated with hemorrhagic transformation in patients receiving intravenous thrombolysis

**DOI:** 10.1038/s41598-021-04716-4

**Published:** 2022-01-12

**Authors:** Sang-Hwa Lee, Min Uk Jang, Yerim Kim, So Young Park, Chulho Kim, Yeo Jin Kim, Jong-Hee Sohn

**Affiliations:** 1 Department of Neurology, Chuncheon Sacred Heart Hospital, Hallym University College of Medicine, 77 Sakju-ro, Chuncheon, South Korea; 2 Department of Neurology, Dongtan Sacred Heart Hospital, Hallym University College of Medicine, Hwaseong, South Korea; 3 Department of Neurology, Kangdong Sacred Heart Hospital, Hallym University College of Medicine, Seoul, South Korea; 4 Department of Endocrinology and Metabolism, Kyung Hee University Hospital, Seoul, South Korea; 5 Institutes of New Frontier Research Team, Hallym University, Chuncheon, South Korea

**Keywords:** Neurological disorders, Risk factors

## Abstract

We evaluated the impact of prestroke glycemic variability estimated by glycated albumin (GA) on symptomatic hemorrhagic transformation (SHT) in patients with intravenous thrombolysis (IVT). Using a multicenter database, we consecutively enrolled acute ischemic stroke patients receiving IVT. A total of 378 patients were included in this study. Higher GA was defined as GA ≥ 16.0%. The primary outcome measure was SHT. Multivariate regression analysis and a receiver operating characteristic curve were used to assess risks and predictive ability for SHT. Among the 378 patients who were enrolled in this study, 27 patients (7.1%) had SHT as defined by the Safe Implementation of Thrombolysis in Stroke-Monitoring Study (SHT_SITS_). The rate of SHT_SITS_ was higher in the higher GA group than in the lower GA group (18.0% vs. 1.6%, *p* < 0.001). A higher GA level (GA ≥ 16.0%) significantly increased the risk of SHT_SITS_ (adjusted odds ratio [OR], [95% confidence interval, CI], 12.57 [3.08–41.54]) in the logistic regression analysis. The predictive ability of the GA level for SHT_SITS_ was good (AUC [95% CI]: 0.83 [0.77–0.90], *p* < 0.001), and the cutoff value of GA in SHT was 16.3%. GA was a reliable predictor of SHT after IVT in acute ischemic stroke in this study.

## Introduction

Hemorrhagic transformation (HT) is a devastating complication that leads to severe disability and high mortality after intravenous thrombolysis (IVT) in acute ischemic stroke patients^1–3^. The occurrence rates of symptomatic HT (SHT) after IVT ranged from 2.4 to 8.8%, and the rates of asymptomatic HT after IVT ranged from 4.5 to 39.6% in several major clinical trials^4^. Because the use of IVT has increased, the rate of HT after IVT may also be increased^5^.

The pathophysiology of HT is disruption of the cerebral microvasculature by acute ischemic events^4^. Several previous studies showed that chronic hyperglycemia estimated by glycated hemoglobin (HbA1c) increased the risk of HT and poor functional outcomes after IVT by inducing microvascular injury^6–9^. A recent experimental study and clinical studies showed that glycemic variability led to more oxidative stress and microvascular injury than chronic hyperglycemia^10–13^. If glycemic variability prior to stroke worsens microvascular injury, then exploring the role of glycemic variability in acute ischemic stroke treated with IVT in clinical practice is important. However, evidence demonstrating whether glycemic variability increases HT and functional outcomes after IVT is sparse.

Glycated albumin (GA) reflects glycemic variability within 4 weeks prior to stroke and may be a useful marker for predicting recent prestroke glycemic variability^14,15^. Using a multicenter registry database, we investigated the effects of glycemic variability estimated by GA on SHT and functional outcomes in patients treated with IVT.

## Results

### Study subjects

Among the 2894 consecutive patients with acute ischemic stroke, 401 patients (13.9%) received recombinant tissue plasminogen activator (tPA) during the study period. Of the 401 patients, 378 patients who met the inclusion criteria were enrolled. Of those 378 patients, 128 patients (33.9%) were in the higher GA group (GA ≥ 16.0%). To perform subgroup analysis, we stratified the 239 of the 378 patients (63.2%) who received only IVT. The higher GA group was more likely to be older, female, have hypertension, diabetes mellitus (DM) and higher initial random glucose, fasting blood glucose and HbA1c levels than the lower GA group. The stroke mechanism (TOAST), acute stroke management and tPA dose were not different between the higher GA and lower GA groups (Table 1). Twenty-seven of the 378 enrolled patients (7.1%) had SHT_SITS_, and 48 patients (12.7%) had any HT. The baseline characteristics of patients receiving IVT only and combined IVT and EVT are described in Supplementary Tables S1 and S2.

**Table 1:** Baseline characteristics of the population according to GA level.

	GA < 16.0% (n = 250)	GA ≥ 16.0% (n = 128)	*p*-value
Age (SD)	64.9 (13.6)	71.5 (11.3)	0.02^a^
Male (%)	163 (65.2)	68 (53.1)	0.03*
BMI, kg/m^2^ (SD)	24.0 (3.4)	23.9 (3.5)	0.82^a^
Interval from onset to IVT, hour (SD)	2.9 (1.01)	3.1 (7.2)	0.81^a^
NIHSS, (IQR)	9 (5–15)	10 (6–15)	0.71^b^
Previous Stroke (%)	43 (17.2)	26 (20.3)	0.48*
Hypertension (%)	112 (44.8)	79 (61.7)	0.002*
DM (%)	25 (10.0)	69 (53.9)	< 0.001*
Hyperlipidemia (%)	32 (12.8)	20 (15.6)	0.53*
Current smoking (%)	44 (17.6)	15 (11.7)	0.18*
Atrial fibrillation (%)	75 (30.0)	50 (39.1)	0.09*
Prior antithrombotic agents (%)	63 (25.2)	41 (32.0)	0.18*
**Stroke mechanism (%)**			0.23*
SVO	28 (11.2)	16 (12.5)	
LAA	75 (30.0)	37 (28.9)	
CE	81 (32.4)	52 (40.6)	
Others	66 (26.4)	52 (18.0)	
**Reperfusion therapy (%)**			0.22*
IVT	164 (65.6)	75 (58.6)	
Combined IVT & IAT	86 (34.4)	53 (41.4)	
**tPA dose (%)**			0.26*
0.6 mg/kg	90 (36.0)	54 (42.2)	
0.9 mg/kg	160 (64.0)	74 (57.8)	
**Lesions (%)**			0.59*
Anterior	202 (80.8)	98 (76.6)	
Posterior	27 (10.8)	18 (14.1)	
Multiple	21 (8.4)	12 (9.4)	
**Vessel occlusion (%)**			0.87*
MCA	145 (86.3)	81 (82.7)	
ICA	3 (1.8)	3 (3.1)	
BA and VA	18 (10.7)	13 (13.3)	
Hemoglobin, mg/dL (SD)	13.8 (2.3)	13.6 (2.0)	0.60^a^
LDL, mg/dL (SD)	104.0 (37.2)	94.0 (38.1)	0.89^a^
Platelet count, × 1000/µL (SD)	237.3 (86.8)	219.5 (61.9)	0.14^a^
Prothrombin time, INR (SD)	1.03 (0.12)	1.04 (0.10)	0.84^a^
Creatinine, mg/dL (SD)	0.98 (0.69)	0.99 (0.10)	0.97^a^
Initial random glucose, mg/dL (SD)	125.7 (37.5)	154.1 (55.3)	< 0.001^a^
Fasting blood glucose, mg/dL (SD)	124.5 (39.3)	152.3 (54.0)	< 0.001^a^
HbA1c, % (SD)	5.7 (0.8)	6.8 (1.2)	< 0.001^a^
Systolic blood pressure, mmHg (SD)	147.8 (26.6)	153.9 (28.8)	0.15^a^
Infarct volume, cm^3^ (IQR)	2.80 (3.39–21.41)	2.98 (9.0–24.9)	0.28^b^

*GA* glycated albumin, *SD* standard deviation, *BMI* body mass index, *NIHSS* national institute health of stroke scale, *IQR* interquartile range, *DM* diabetes mellitus, *SVO* small vessel occlusion, *LAA* large artery atherosclerosis, *CE* cardioembolism, *IVT* intravenous thrombolysis, *IAT* intraarterial thrombectomy, *tPA* tissue plasminogen activator, *MCA* middle cerebral artery, *ICA* internal carotid artery, *BA* basilar artery, *VA* vertebral artery, *LDL* low density lipoprotein, *INR* international normalized ratio, HbA1c, glycated hemoglobin.

*Calculated using the chi-square test.

^a^Calculated using Student’s t-test.

^b^Calculated using the Mann–Whitney U test.

### Rates of SHT

**Figure 1:**
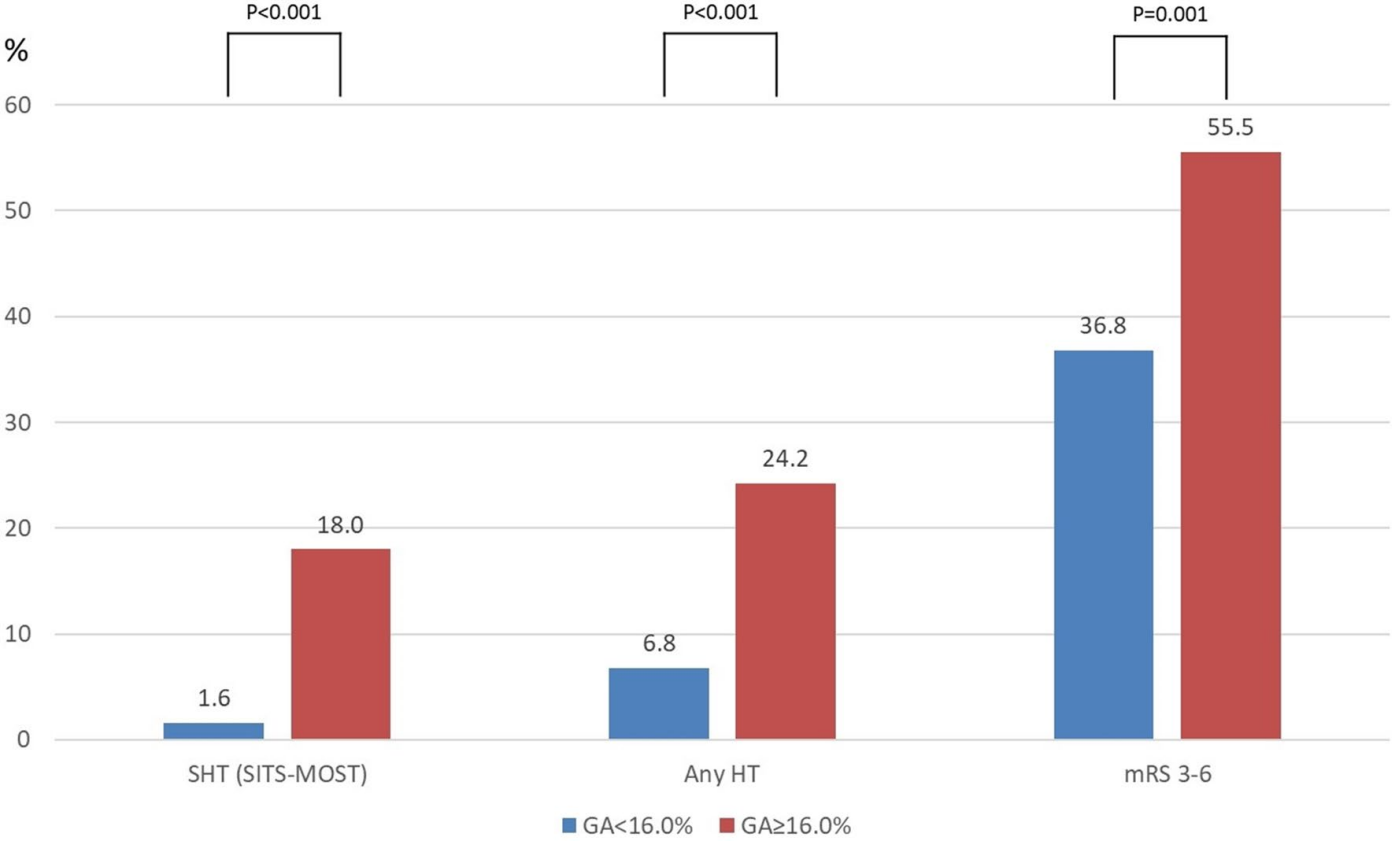
Distributions of SHT and mRS scores 3–6 according to GA levels. Abbreviations: SHT, symptomatic hemorrhagic transformation; mRS, modified Rankin Scale; GA, glycated albumin; SITS-MOST, Safe Implementation of Thrombolysis in Stroke-Monitoring Study; ECASS II, European-Australian Cooperative Acute Stroke Study II; HT, hemorrhagic transformation.

The rate of SHT_SITS_ was higher in the higher GA group than in the lower GA group (SHT_SITS_: 18.0% vs. 1.6%, *p* < 0.001, Fig. 1). The rate of any HT was also higher in the higher GA group than in the lower GA group (24.2% vs. 6.8%, *p* < 0.001, Fig. 1). The proportion of individuals who had poor functional outcomes at 3 months (mRS score of 3–6) was higher in the higher GA group than in the lower GA group (55.5% vs. 36.8%, *p* = 0.001, Fig. 1). The rates of SHT in the patients receiving IVT only and combined IVT and EVT were higher in the higher GA group (1.8% vs. 12.0% in the IVT only group; 1.2% vs. 26.4% in the combined IVT and EVT group, Supplementary Tables S1 and S2 online).

### GA level and risk of SHT

The logistic regression analysis showed that a higher GA level (GA ≥ 16.0%) significantly increased the risk of SHT_SITS_ (adjusted OR, [95% CI], 12.57 [3.08–41.54], Table 2 and see Supplementary Table S3 online). A higher GA level also increased the risk of any HT (adjusted OR, [95% CI], 5.50 [2.60–11.66], Table 2 and see Supplementary Table S3 online). A higher GA level also increased the risk of SHT_SITS_ in both separate databases in the subgroup analysis (Supplementary Tables S4 and S5 online). For the secondary outcome measures, a higher GA level (GA ≥ 16.0%) was significantly associated with poor functional outcomes at 3 months.

**Table 2:** Logistic regression showing the impact of GA and GA/HbA1c ratio on SHT and poor functional outcome (mRS 3–6).

	SHT_SITS_		Any HT		mRS 3–6	
	aOR	95% CI	aOR	95% CI	aOR	95% CI
GA ≥ 16.0%	12.57	3.08–41.54	5.50	2.60–11.66	2.16	1.21–3.85
GA/HbA1c	3.39	1.26–9.16	2.16	1.01–4.61	2.03	1.08–3.80
raw GA	1.29	1.11–1.50	1.25	1.11–1.41	1.13	1.03–1.25

*GA* glycated albumin, HbA1c, glycated hemoglobin; *SHT* symptomatic hemorrhagic transformation, *mRS* modified rankin scale, *SHT*_SITS_ symptomatic hemorrhagic transformation defined per safe implementation of thrombolysis in stroke-monitoring study, *aOR* adjusted odds ratio, *CI* confidence interval.

### Predictive ability of GA on SHT

**Figure 2:**
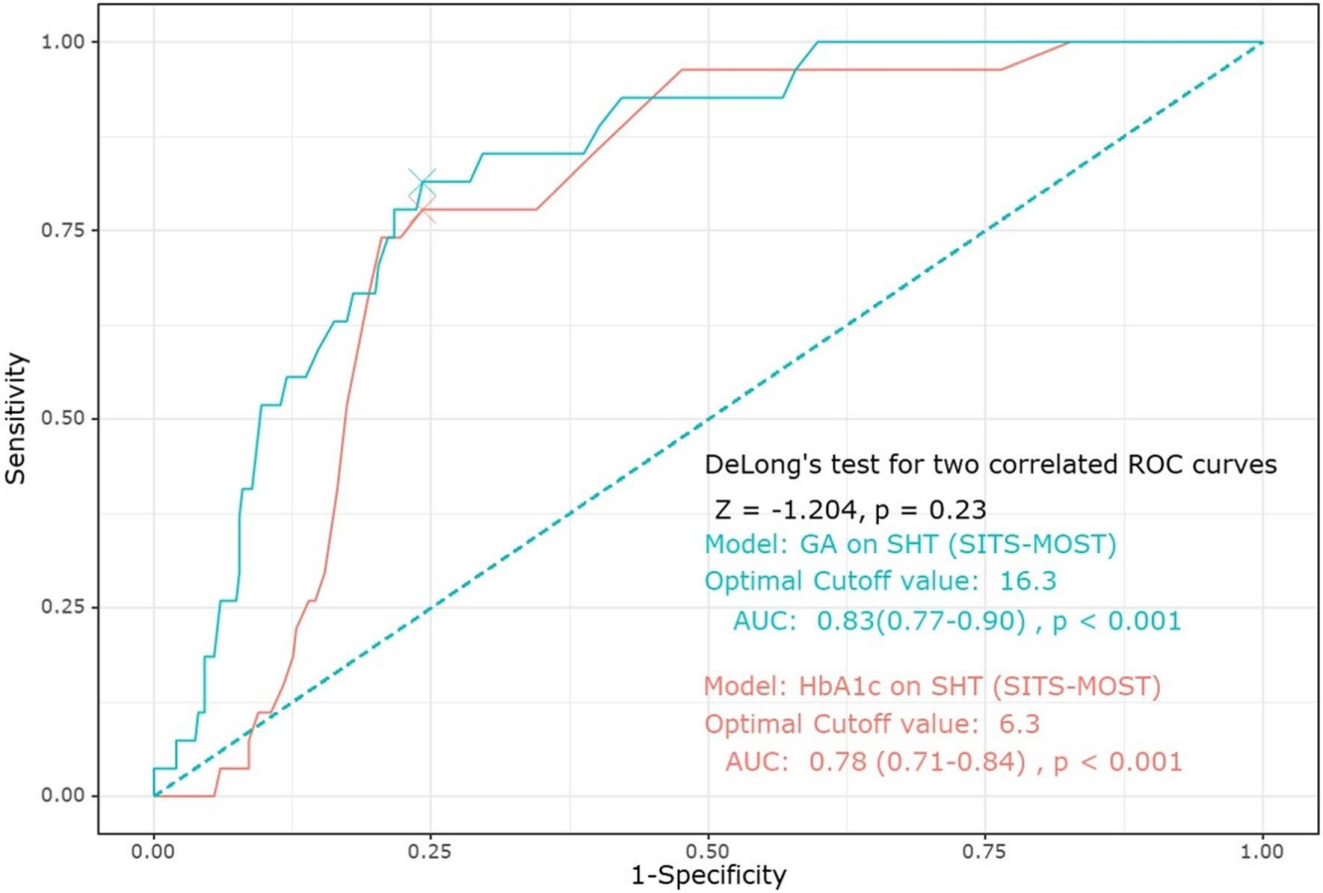
ROC curve showing the predictive ability and cutoff points of GA and HbA1c levels for SHT. Abbreviations: ROC, receiver operating characteristic; GA, glycated albumin; HbA1c, glycated hemoglobin; SHT, symptomatic hemorrhagic transformation; SITS-MOST; AUC, area under the curve.

The receiver operating characteristic (ROC) curve showed that the predictive ability of GA and HbA1c levels for SHT_SITS_ was close to good (AUC of GA: 0.83, 95% CI [0.77–0.90], *p* < 0.001; AUC of HbA1c: 0.78, 95% CI [0.71–0.84], *p* < 0.001). However, there were no significant differences in the prediction of SHT_SITS_ between GA and HbA1c levels (Fig. 2). The cutoff values of GA and HbA1c were 16.3% and 6.3%, respectively, for SHT_SITS_. For patients who received only IVT, the predictive ability of GA and HbA1c levels for SHT_SITS_ was close to good (AUC of GA: 0.77, 95% CI [0.65–0.89], *p* < 0.001; AUC of HbA1c, 95% CI [0.62–0.85], *p* < 0.001). The cutoff values of GA and HbA1c were 16.0% and 6.3%, respectively, for SHT_SITS_ (see Supplementary Fig. S1 online).

### Sensitivity analysis

Sensitivity analysis revealed that the GA/HbA1c ratio and raw GA significantly increased the risk of SHT_SITS_ and any HT. GA/HbA1c was significantly associated with poor functional outcomes at 3 months (see Supplementary Tables S6 and S7 online). When we stratified the patients by the presence of DM, a higher GA level significantly increased the risk of any HT, and the GA/HbA1c ratio significantly increased the risk of SHT_SITS_ and any HT (see Supplementary Tables S8 and S9 online). A higher HbA1c level also increased the risk of SHT_SITS_ and any HT but not poor functional outcomes (see Supplementary Table S10 online).

## Discussion

The main findings of the present study are as follows: (1) The higher GA group had more occurrences of SHT and poor functional outcomes in acute ischemic stroke patients receiving IVT. (2) GA was a strong independent marker for predicting SHT and poor functional outcomes after receiving IVT. (3) The predictive ability of GA for SHT was reliable and the cutoff value of GA was 16.3%.

Glycemic variability exacerbates the breakdown of the blood–brain barrier via oxidative stress and aggravates microangiopathy, which induces HT after IVT^13,16,17^. Therefore, we hypothesized that GA, which measures recent prestroke glycemic variability quickly and easily in an acute stroke setting, would be a reliable parameter for predicting postthrombolytic bleeding. This multicenter cohort study is the first to evaluate the association between GA and SHT in patients receiving IVT, and the results support this hypothesis. The rates of SHT_SITS_ in our study were comparable to those in previous clinical studies^4,18^, and a high GA level increased the risk of SHT_SITS_ and any HT. The sensitivity analysis revealed that the impact of the GA/HbA1c ratio and GA in patients treated with IVT and DM on SHT improved the robustness of our results. We also showed that GA and HbA1c increased the risk of SHT (see Supplementary Table S10 online), which is consistent with previous studies. GA may be useful to predict SHT after IVT in real-world practice.

Our study revealed the predictive ability and cutoff point of GA for SHT after IVT. The ROC curve analysis showed that GA and HbA1c were reliable for predicting SHT_SITS_ after IVT, but GA was not superior to HbA1c in predicting SHT_SITS_. Notably, the best cutoff points for predicting SHT_SITS_ were 16.3% and 6.3% with GA and HbA1c, respectively. This result is the novel finding of our study. The cutoff point for predicting SHT with HbA1c (6.3%) in our study was comparable to that in a previous study (cutoff point of HbA1c, 6.5%)^6^. We identified that GA and HbA1c levels may be useful markers for predicting SHT in patients receiving IV tPA using multivariate analysis and ROC curves.

A high GA level was associated with poor functional outcomes at 3 months in our study, but a high HbA1c level was not. Recent studies showed that high HbA1c was an independent predictor of 3-month functional outcomes^8,19,20^. However, the association between HbA1c and functional outcomes remains controversial^21,22^. In contrast, previous clinical studies on prestroke glycemic variability showed consistent results, and glycemic variability was associated with poor outcomes in stroke patients^11,23^. Similar to previous studies, we showed that a high GA level increased the risk of poor functional outcomes in patients treated with IVT. This result may be explained by the high incidence of SHT in the high GA group, despite the similarity in infarct volume between the two groups^3^. The SHT group in this study had greater stroke severity and eventually had a high proportion of poor functional outcomes (see Supplementary Table S11 online). The exact mechanism of the association of glycemic control with poor functional outcomes is not clear. Future studies are warranted to clarify the association between glycemic variability and poor functional outcomes.

Notably, blood glucose levels on admission (initial random glucose and fasting blood glucose) were not associated with SHT in our multivariate analysis, which is different from several studies^24,25^. Previous studies found that prestroke glycemic control was not associated with poststroke blood glucose levels^26^. These findings suggest that prestroke glycemic control is more closely related to predicting SHT than poststroke glucose levels. The traditional method for measuring glycemic variability is to estimate the standard deviation (SD) and coefficient of variation of the mean poststroke blood glucose level. If the poststroke glucose level is not reliable for predicting SHT after IVT, then the clinical use of the GA test, which estimates prestroke glycemic variability easily with one blood sample, may be used in an acute stroke setting.

Our study has several limitations despite the use of a multicenter database. First, although we collected data consecutively, it was an observational retrospective study. Second, we cannot ensure the exclusion of unmeasured confounding variables, but we adjusted for several confounders in the statistical analyses. Third, due to the small sample size of subjects with IVT only, the heterogeneity of recanalization therapies (e.g., use of tPA doses and addition of EVT) in our study may hinder the generalization of our results. However, the subgroup analysis evaluating subjects treated with only IVT showed that higher GA was also associated with SHT. This finding improves the robustness of our main result. Fourth, we did not consider several medical conditions that increase protein metabolism and affect GA levels. Thyroid function, liver function, renal dysfunction and alcohol consumption were not available in our database. However, GA was measured on a routine basis prospectively at the two stroke centers, and missing GA data were uncommon. This measurement is a strength of our study.

In conclusion, the results of our study showed that prestroke glycemic variability estimated by GA is associated with SHT and poor functional outcomes in patients treated with IVT. Because the GA level is measured quickly and is a reliable marker for predicting SHT, monitoring prestroke glycemic variability via GA levels may be helpful to establish strategies for the prevention of SHT and poor functional outcomes in acute stroke patients receiving IVT.

## Methods

### Subjects

We consecutively registered all acute ischemic stroke patients between March 2016 and December 2020 at two university-affiliated institutions (Chuncheon Sacred Heart Hospital and Dongtan Sacred Heart Hospital). We identified acute ischemic stroke patients treated with IVT. All of the included patients received intravenous recombinant tPA according to current guidelines for acute stroke management. We excluded the following patients: (1) patients with unavailable GA measurements; (2) patients without follow-up brain computed tomography (CT) or magnetic resonance imaging (MRI) within 24 h of stroke onset; (3) patients who were not available for assessment with the National Institutes of Health Stroke Scale (NIHSS) after IVT and the modified Rankin Scale (mRS) at 3 months; and (4) patients with a prestroke mRS score ≥ 2.

### Ethics declaration

This retrospective cohort study was conducted in accordance with the Helsinki declaration and approved by the Institutional Review Board (IRB) of the Hallym University Chuncheon Sacred Heart Hospital (IRB number: 2021-03-002) and Hallym University Dongtan Sacred Heart Hospital (IRB number: 2021-03-006). The need for written informed consent was waived and confirmed by the IRB of Hallym University Chuncheon Sacred Heart Hospital (IRB number: 2021-03-002) and Hallym University Dongtan Sacred Heart Hospital (IRB number: 2021-03-006) because this was a retrospective cohort study.

### Data collection and definition of parameters

The following data were directly obtained from the registry database: (1) demographics, including age and sex; (2) stroke risk factors, medical history, prior stroke, the presence of hypertension, DM, hyperlipidemia, atrial fibrillation, current smoking status, prestroke status, and prior use of statins and antithrombotic drugs; (3) stroke characteristics, acute stroke treatment, initial NIHSS score, ischemic stroke mechanism according to the Trial of Org 10,172 in Acute Stroke Treatment (TOAST) classification, tPA dose and reperfusion therapy (IVT and intra-arterial thrombectomy [IAT]), modified Thrombolysis In Cerebral Infarction (mTICI) grade 2b to 3 after IAT^27^; and (4) laboratory data, such as platelet count, prothrombin time, systolic blood pressure and level of hemoglobin, creatinine, initial random glucose, fasting blood glucose, low-density lipoprotein and HbA1c. The infarct volume confirmed by diffusion weighted imaging was calculated using the ABC/2 method^28^.

The primary outcome measure was the occurrence of SHT. SHT was defined according to the Safe Implementation of Thrombolysis in Stroke-Monitoring Study (SITS-MOST): type 2 parenchymal hemorrhage with deterioration in the NIHSS score of ≥ 4 points or death (SHT_SITS_)^29^. Two trained neurologists (S–H Lee and MU Jang) reviewed the CT/MR images to confirm the subtypes of HT in a double-blinded manner. The secondary outcome measures were any HT and poor functional outcomes assessed by a mRS score of 3–6 at 3 months^30^. Any HT was defined as any newly developed bleeding on the follow-up CT or MRI after IVT.

Recent previous studies established that a GA level ≥ 16.0% reflected the presence of prestroke glycemic variability based on the following equation of HbA1c and GA: HbA1c = 0.216 × GA + 2.978^11,23,31^. According to this equation, we divided the subjects into two groups: a higher GA group (GA ≥ 16.0%) and a lower GA group (GA < 16.0%). GA may be a useful marker for monitoring prestroke glycemic variability because GA is not affected by several medical conditions (hematologic disorder, chronic renal disease, etc.), which account for a large proportion of stroke patients^32^. All procedures were conducted in accordance with the STROBE guidelines.

### Statistical analysis

We hypothesized that high glycemic variability prior to stroke, as defined by a higher GA level, would increase the risk of SHT and poor functional outcomes after IVT. Statistical analyses were performed using IBM SPSS version 21.0 software (IBM Corporation, Armonk, NY, USA) and R version 4.0.3 (R core Team 2020; R foundation for Statistical Computing, Vienna, Austria). Summary statistics are presented as the number of subjects (percentage) for categorical variables and the means ± SD or median [interquartile range (IQR)] for continuous variables. Group comparisons were made using Pearson’s chi-squared test for categorical variables and Student’s t-test or the Mann–Whitney U test for continuous variables, as appropriate.

For the primary and secondary outcome measures, the higher GA group and lower GA group were compared using Pearson’s chi-squared test for categorical variables and Student’s t-test or the Mann–Whitney U test for continuous variables. To evaluate the independent effects of GA level on outcome measures, we performed binary logistic regression analysis. Variables for adjustment in the multivariable analysis were selected when their p values were < 0.1 in comparisons according to the GA level and if their associations with each outcome variable were clinically plausible. Crude and adjusted ORs and 95% CIs were estimated.

To assess the predictive ability of GA and HbA1c levels on SHT, we generated an ROC curve using the ‘pROC’ package of R. The 95% CI for the area under the curve (AUC) and *p* value was calculated using Delong’s test. The cutoff values of GA and HbA1c levels for SHT were calculated using the Youden index.

A sensitivity analysis was also conducted to explore the impact of the GA to HbA1c ratio (GA/HbA1c ratio) and raw GA level on outcomes using a logistic regression model because the GA/HbA1c ratio represented a more accurate glycemic control status. For subgroup analyses, we performed the same statistical models to explore the impact of GA on outcomes in patients receiving IVT only and combined IVT and EVT separately and in those with DM. DM patients were defined as patients with high HbA1c levels (≥ 6.5%) at the time of hospitalization or patients with a history of or current use of hypoglycemic agents or insulin.

## Supplementary Information

Supplementary Information.

## Data Availability

All datasets generated and/or analyzed during the current study are not publicly available as use of the data requires ethical approval. To inquire access to the study data, contact the corresponding author.
