# Supplementary material for: High glycemic albumin representing prestroke glycemic variability is associated with hemorrhagic transformation in patients receiving intravenous thrombolysis
Source: Sci Rep. 2022 Jan 12;12:615. doi: 10.1038/s41598-021-04716-4 (PMC8755722; doi:10.1038/s41598-021-04716-4)

**Supplement materials**

**Supplemental Table S1**. Baseline characteristics of the population according to GA level in patients receiving IVT only

|  | GA <16.0%  (n=164) | GA≥16.0%  (n=75) | p-value |
| --- | --- | --- | --- |
| Age (SD) | 64.2 (13.5) | 70.4 (11.2) | 0.04^†^ |
| Male (%) | 110 (67.1) | 39 (52.0) | 0.03^*^ |
| BMI, kg/m^2^ (SD) | 24.0 (12.4) | 23.9 (9.4) | 0.95^†^ |
| Interval from  onset to IVT, hour (SD) | 3.3 (12.4) | 4.0 (9.4) | 0.68^†^ |
| NIHSS, (IQR) | 7 (5-11) | 7 (5-12) | 0.68^‡^ |
| Previous Stroke (%) | 29 (17.7) | 16 (21.3) | 0.50^*^ |
| Hypertension (%) | 75 (45.7) | 47 (62.7( | 0.02^*^ |
| DM (%) | 19 (11.6) | 45 (60.0) | <0.001^*^ |
| Hyperlipidemia (%) | 18 (11.0) | 13 (17.3) | 0.18^*^ |
| Current smoking (%) | 32 (19.5) | 10 (13.3) | 0.24^*^ |
| Atrial fibrillation (%) | 39 (23.8) | 21 (28.0) | 0.49^*^ |
| Prior antithrombotic agents (%) | 38 (23.2) | 20 (26.7) | 0.63^*^ |
| Stroke mechanism (%) |  |  | 0.31^*^ |
| SVO | 28 (17.1) | 16 (21.3) |  |
| LAA | 50 (30.5) | 26 (34.7) |  |
| CE | 39 (23.8) | 20 (26.7) |  |
| Others | 47 (28.7) | 13 (17.3) |  |
| tPA dose (%) |  |  | 0.27^*^ |
| 0.6 mg/kg | 62 (37.8) | 34 (45.3) |  |
| 0.9 mg/kg | 102 (62.2) | 41 (54.7) |  |
| Hemoglobin, mg/dL (SD) | 13.8 (2.4) | 13.8 (1.9) | 0.32^†^ |
| LDL, mg/dL (SD) | 107.5 (36.5) | 99.0 (39.2) | 0.67^†^ |
| Platelet count, ×1000/µL (SD) | 242.4 (90.1) | 225.1 (57.7) | 0.20^†^ |
| Prothrombin time, INR (SD) | 1.03 (0.11) | 1.04 (0.10) | 0.50^†^ |
| Creatinine, mg/dL (SD) | 1.02 (0.82) | 0.96 (0.36) | 0.26^†^ |
| Initial random glucose, mg/dL (SD) | 125.8 (39.8) | 153.6 (56.9) | <0.001^†^ |
| Fasting blood glucose, mg/dL (SD) | 124.9 (43.9) | 153.2 (53.9) | 0.01^†^ |
| HbA1c, % (SD) | 5.8 (1.0) | 6.6 (1.2) | <0.001^†^ |
| Systolic blood pressure, mmHg (SD) | 148.2 (26.0) | 156.2 (29.1) | 0.19^†^ |
| Infarct volume, cm^3^ (IQR) | 1.29 (1.22-6.50) | 1.33 (7.10-15.20) | 0.08^‡^ |
| Outcomes |  |  |  |
| SHT_SITS_ | 3 (1.8) | 9 (12.0) | 0.001^*^ |
| 3-month mRS 3 to 6 | 50 (30.5) | 37 (49.3) | 0.01^*^ |

Abbreviations: GA, glycated albumin; IVT, intravenous thrombolysis; SD, standard deviation; BMI, body mass index; NIHSS, National Institute Health of Stroke Scale; IQR, interquartile range; DM, diabetes mellitus; SVO, small vessel occlusion; LAA, large artery atherosclerosis; CE, cardioembolism; tPA, tissue plasminogen activator; LDL, low density lipoprotein; INR, international normalized ratio; HbA1c, glycated hemoglobin; SHT_SITS_: Symptomatic hemorrhagic transformation; mRS. Modified Rankin Scale

^*^ Calculated using the chi-square test

^†^ Calculated using Student’s t-test

^‡^ Calculated using the Mann-Whitney U test

**Supplemental Table S2**. Baseline characteristics of the population according to GA level in patients receiving combined IVT and EVT.

|  | GA <16.0%  (n=164) | GA≥16.0%  (n=75) | p-value |
| --- | --- | --- | --- |
| Age (SD) | 68.4 (13.7) | 73.0(11.3) | 0.20^†^ |
| Male (%) | 53 (61.6) | 29 (54.7) | 0.48^*^ |
| BMI, kg/m^2^ (SD) | 24.0 (3.4) | 23.8 (3.4) | 0.64^†^ |
| Interval from  onset to IVT, hour (SD) | 2.07 (1.72) | 1.92 (1.13) | 0.76^†^ |
| Interval from  onset to reperfusion, hour (SD) | 2.41 (1.92) | 2.65 (3.25) | 0.29^†^ |
| NIHSS, (IQR) | 15 (11-17) | 14 (10-18) | 0.71^‡^ |
| Previous Stroke (%) | 14 (16.3) | 10 (18.9) | 0.82^*^ |
| Hypertension (%) | 37 (43.0) | 32 (60.4) | 0.06^*^ |
| DM (%) | 6 (7.0) | 24 (45.3) | <0.001^*^ |
| Hyperlipidemia (%) | 14 (16.3) | 7 (13.2) | 0.64^*^ |
| Current smoking (%) | 12 (14.0) | 5 (9.4) | 0.60^*^ |
| Atrial fibrillation (%) | 36 (41.9) | 29 (54.7) | 0.16^*^ |
| Prior antithrombotic agents (%) | 25 (29.1) | 21 (39.6) | 0.27^*^ |
| Stroke mechanism (%) |  |  | 0.40^*^ |
| LAA | 26 (29.1) | 11 (20.8) |  |
| CE | 42 (48.8) | 32 (60.4) |  |
| Others | 19 (22.1) | 10 (18.9) |  |
| tPA dose (%) |  |  | 0.58^*^ |
| 0.6 mg/kg | 58 (67.4) | 33 (62.3) |  |
| 0.9 mg/kg | 28 (32.6) | 20 (37.7) |  |
| Hemoglobin, mg/dL (SD) | 13.7 (2.1) | 13.3 (2.1) | 0.60^†^ |
| LDL, mg/dL (SD) | 97.3 (37.9) | 86.8(35.6) | 0.89^†^ |
| Platelet count, ×1000/µL (SD) | 227.6 (79.7) | 211.6 (67.1) | 0.41^†^ |
| Prothrombin time, INR (SD) | 1.04 (0.14) | 1.06 (0.11) | 0.71^†^ |
| Creatinine, mg/dL (SD) | 0.90 (0.30) | 1.04 (0.46) | 0.07^†^ |
| Initial random glucose, mg/dL (SD) | 12.5 (32.7) | 154.8 (53.5) | 0.001^†^ |
| Fasting blood glucose, mg/dL (SD) | 123.3 (28.8) | 151.2 (54.7) | <0.001^†^ |
| HbA1c, % (SD) | 5.6 (0.6) | 6.9 (1.3) | <0.001^†^ |
| Systolic blood pressure, mmHg (SD) | 147.0 (24.4) | 150.8 (28.4) | 0.64^†^ |
| Infarct volume, cm^3^ (IQR) | 11.22 (2.70-38.91) | 4.91 (2.11-29.15) | 0.30^‡^ |
| Vessel occlusion site |  |  | 0.80^*^ |
| MCA | 79 (91.9) | 48 (90.6) |  |
| ICA | 3 (3.5) | 1 (1.9) |  |
| BA and VA | 4 (4.7) | 4 (7.5) |  |
| mTICI grade 2b to 3 | 70 (81.4) | 42 (79.2) | 0.83^*^ |
| Outcomes |  |  |  |
| SHT_SITS_ | 1 (1.2) | 14 (26.4) | <0.001^*^ |
| 3-month mRS 3 to 6 | 42 (48.8) | 34 (64.2) | 0.08^*^ |

Abbreviations: GA, glycated albumin; IVT, intravenous thrombolysis; SD, standard deviation; BMI, body mass index; NIHSS, National Institute Health of Stroke Scale; IQR, interquartile range; DM, diabetes mellitus; LAA, large artery atherosclerosis; CE, cardioembolism; tPA, tissue plasminogen activator; LDL, low density lipoprotein; INR, international normalized ratio; HbA1c, glycated hemoglobin; MCA, middle cerebral artery; ICA, internal carotid artery; BA, basilar artery; VA, vertebral artery; mTICI, modified Thrombolysis in Cerebral infarction; SHT_SITS_, Symptomatic hemorrhagic transformation; mRS, Modified Rankin Scale

^*^ Calculated using the chi-square test

^†^ Calculated using Student’s t-test

^‡^ Calculated using the Mann-Whitney U test

**Supplemental Table S3.** Logistic regression analysis showing multiple covariates including GA≥16% on outcomes.

|  | SHT_SITS_ | | | Any HT | | | mRS 3-6 | | |
| --- | --- | --- | --- | --- | --- | --- | --- | --- | --- |
|  | aOR | 95% CI | p-value | aOR | 95% CI | p-value | aOR | 95% CI | p-value |
| GA≥16% | 12.57 | 3.80-41.54 | <0.001 | 5.50 | 2.60-11.66 | <0.001 | 2.16 | 1.21-3.85 | 0.01 |
| Age | 1.003 | 0.96-1.05 | 0.88 | 0.997 | 0.97-1.03 | 0.83 | 1.01 | 1.99-1.03 | 0.42 |
| Male | 0.71 | 0.27-1.84 | 0.48 | 0.53 | 0.30-1.25 | 0.18 | 0.69 | 0.42-1.14 | 0.15 |
| NIHSS per 1 point | 1.12 | 1.03-1.21 | 0.01 | 1.05 | 0.98-1.11 | 0.15 | 1.19 | 1.13-1.25 | <0.001 |
| TOAST |  |  |  |  |  |  |  |  |  |
| SVO |  | reference |  |  | reference |  |  | reference |  |
| LAA | 1.22 | 0.20-7.24 | 0.83 | 1.65 | 0.46-5.85 | 0.44 | 1.64 | 0.68-3.97 | 0.27 |
| CE | 0.75 | 0.09-6.32 | 0.8 | 0.62 | 0.12-3.24 | 0.57 | 0.70 | 0.21-2.32 | 0.56 |
| Others | 1.37 | 0.21-9.19 | 0.74 | 0.82 | 0.20-3.37 | 0.79 | 1.06 | 0.41-2.71 | 0.91 |
| HTN | 0.85 | 0.32-2.23 | 0.74 | 0.85 | 0.42-1.73 | 0.66 | 1.22 | 0.74-2.02 | 0.44 |
| DM | 0.86 | 0.32-2.35 | 0.77 | 0.42 | 0.18-1.002 | 0.051 | 0.89 | 0.74-1.68 | 0.72 |
| A.fib | 1.60 | 0.39-6.57 | 0.52 | 2.46 | 0.73-8.27 | 0.15 | 1.54 | 0.60-3.95 | 0.37 |
| IRG | 1.01 | 0.996-1.02 | 0.22 | 1.004 | 0.99-1.01 | 0.41 | 0.998 | 0.99-1.01 | 0.67 |
| FBG | 0.999 | 0.99-1.01 | 0.86 | 0.999 | 0.99-1.01 | 0.86 | 1.002 | 0.995-1.01 | 0.58 |
| SBP | 1.01 | 0.996-1.03 | 0.16 | 1.004 | 0.99-1.02 | 0.49 | 1.01 | 0.998-1.02 | 0.11 |

**Supplementary Table S4.** Logistic regression analysis showing the impact of multiple variables, including GA, on outcomes in patients receiving IVT only.

|  | SHT_SITS_ | | | Any HT | | | mRS 3-6 | | |
| --- | --- | --- | --- | --- | --- | --- | --- | --- | --- |
|  | aOR | 95% CI | p-value | aOR | 95% CI | p-value | aOR | 95% CI | p-value |
| GA≥16% | 8.08 | 1.39-46.97 | 0.02 | 4.00 | 1.43-11.20 | 0.01 | 2.45 | 1.13-5.33 | 0.02 |
| Age | 1.09 | 1.01-1.18 | 0.04 | 1.02 | 0.98-1.07 | 0.34 | 1.01 | 0.98-1.04 | 0.59 |
| Male | 0.54 | 0.11-2.71 | 0.46 | 0.72 | 0.28-1.86 | 0.50 | 0.99 | 0.51-1.91 | 0.97 |
| NIHSS per 1 point | 1.17 | 1.03-1.34 | 0.02 | 1.06 | 0.97-1.15 | 0.23 | 1.24 | 1.15-1.34 | <0.001 |
| TOAST |  |  |  |  |  |  |  |  |  |
| SVO |  | reference |  |  | reference |  |  | reference |  |
| LAA | 0.53 | 0.06-4.70 | 0.57 | 1.13 | 0.28-4.50 | 0.87 | 1.52 | 0.59-3.94 | 0.39 |
| CE | 0.29 | 0.03-3.21 | 0.31 | 1.15 | 0.28-5.36 | 0.86 | 1.38 | 0.48-4.02 | 0.55 |
| Others | 2.15 | 0.23-19.85 | 0.50 | 0.77 | 0.16-3.66 | 0.75 | 1.01 | 0.36-2.85 | 0.98 |
| HTN | 0.53 | 0.12-2.32 | 0.40 | 0.68 | 0.26-1.76 | 0.43 | 1.03 | 0.54-1.98 | 0.93 |
| DM | 2.39 | 0.50-11.49 | 0.28 | 0.50 | 0.16-1.58 | 0.24 | 0.89 | 0.74-1.68 | 0.72 |
| IRG per 1 mg/dL | 1.02 | 1.002-1.03 | 0.03 | 1.01 | 0.99-1.02 | 0.43 | 0.998 | 0.99-1.01 | 0.67 |
| FBS per 1 mg/dL | 0.99 | 0.97-1.01 | 0.23 | 0.996 | 0.98-1.01 | 0.58 | 1.002 | 0.995-1.01 | 0.58 |
| SBP per 1 mmHg | 1.02 | 0.995-1.05 | 0.11 | 1.001 | 0.98-1.02 | 0.94 | 1.01 | 0.998-1.02 | 0.11 |

IRB, initial random glucose, FBS, fasting blood sugar, SBP, systolic blood pressure

**Supplementary Table S5**. Logistic regression analysis showing multiple covariates including GA≥16% on outcomes in patients receiving combined IVT and EVT.

|  |  | SHT_SITS_ |  |  | Any HT |  |  | mRS 3-6 |  |
| --- | --- | --- | --- | --- | --- | --- | --- | --- | --- |
|  | aOR | 95% CI | p-value | aOR | 95% CI | p-value | aOR | 95% CI | p-value |
| GA≥16% | 22.04 | 1.21-400.33 | 0.04 | 7.70 | 1.66-35.81 | 0.01 | 1.13 | 0.43-4.01 | 0.64 |
| Age | 0.98 | 0.90-1.08 | 0.72 | 0.998 | 0.94-1.06 | 0.94 | 1.03 | 0.99-1.08 | 0.10 |
| Male | 0.98 | 0.13-7.45 | 0.98 | 0.56 | 0.14-.17 | 0.4 | 0.5 | 0.19-1.32 | 0.16 |
| NIHSS per 1 point | 1.02 | 0.861.21 | 0.84 | 1.02 | 0.90-1.16 | 0.75 | 1.14 | 1.04-1.26 | 0.01 |
| TOAST |  |  |  |  |  |  |  |  |  |
| others |  |  |  |  |  |  |  |  |  |
| CE | 1.64 | 0.04-73.22 | 0.8 | 3.25 | 0.32-33.47 | 0.32 | 1.04 | 0.27-4.12 | 0.95 |
| LAA | 19.98 | 0.84-474.34 | 0.06 | 3.47 | 0.50-27.93 | 0.2 | 0.76 | 0.24-2.38 | 0.64 |
| Interval from onset to reperfusion | 0.99 | 0.74-1.32 | 0.95 | 0.92 | 0.66-1.29 | 0.63 | 0.95 | 0.80-1.14 | 0.59 |
| TICI grade 2b to 3 | 0.04 | 0.001-1.14 | 0.06 | 0.42 | 0.05-3.47 | 0.42 | 0.23 | 0.03-1.54 | 0.13 |
| Infarct volume | 1.00 | 0.99-1.00 | 0.51 | 1 | 1.00-1.00 | 0.06 | 1 | 1.00-1.00 | 0.01 |
| DM | 5.24 | 0.65-42.35 | 0.12 | 0.96 | 0.19-4.75 | 0.96 | 1.01 | 0.27-3.82 | 0.99 |
| IRG per 1 mg/dL | 0.96 | 0.92-0.996 | 0.03 | 0.99 | 0.97-1.01 | 0.27 | 0.99 | 0.97-1.01 | 0.25 |
| FBS per 1 mg/dL | 1.03 | 1.00-1.06 | 0.04 | 1.01 | 0.99-1.03 | 0.35 | 1.02 | 0.996-1.04 | 0.11 |
| Creatinine per 1 mg/dL | 50.7 | 1.70-1512.26 | 0.02 | 4.21 | 0.69-25.61 | 0.12 | 1.34 | 0.38-4.71 | 0.65 |

IRB, initial random glucose, FBS, fasting blood sugar, SBP, systolic blood pressure

**Supplemental Table S6.** Logistic regression analysis showing the impact of multiple covariates, including the GA/HbA1c ratio on outcomes.

|  | SHT_SITS_ | | | Any HT | | | mRS 3-6 | | |
| --- | --- | --- | --- | --- | --- | --- | --- | --- | --- |
|  | aOR | 95% CI | p-value | aOR | 95% CI | p-value | aOR | 95% CI | p-value |
| GA/HbA1c ratio | 3.39 | 1.26-9.16 | 0.02 | 2.16 | 1.01-4.61 | 0.046 | 2.03 | 1.08-3.80 | 0.03 |
| Age | 1.01 | 0.97-1.06 | 0.54 | 1.01 | 0.98-1.04 | 0.73 | 1.01 | 0.99-1.03 | 0.25 |
| Male | 0.86 | 0.34-2.18 | 0.75 | 0.66 | 0.33-1.31 | 0.23 | 0.73 | 0.44-1.21 | 0.23 |
| NIHSS per 1 point | 1.11 | 1.03-1.20 | 0.01 | 1.05 | 0.99-1.11 | 0.14 | 1.19 | 1.13-1.25 | <0.001 |
| TOAST |  |  |  |  |  |  |  |  |  |
| SVO |  | reference |  |  | reference |  |  | reference |  |
| LAA | 1.14 | 0.18-7.21 | 0.89 | 1.38 | 0.40-4.80 | 0.61 | 1.52 | 0.63-3.68 | 0.36 |
| CE | 0.77 | 0.09-6.69 | 0.81 | 0.54 | 0.11-2.74 | 0.46 | 0.63 | 0.19-2.10 | 0.45 |
| Others | 1.43 | 0.20-10.10 | 0.72 | 0.76 | 0.19-3.03 | 0.69 | 0.99 | 0.39-2.53 | 0.99 |
| HTN | 0.81 | 0.31-2.08 | 0.66 | 0.86 | 0.43-1.70 | 0.66 | 1.19 | 0.72-1.96 | 0.50 |
| DM | 1.82 | 0.68-4.85 | 0.23 | 0.77 | 0.34-1.74 | 0.53 | 1.13 | 0.63-2.04 | 0.68 |
| A.fib | 1.57 | 0.38-6.54 | 0.54 | 2.48 | 0.75-8.15 | 0.14 | 1.54 | 0.61-3.92 | 0.36 |
| IRG per 1 mg/dL | 1.01 | 0.995-1.02 | 0.24 | 1.01 | 0.996-1.02 | 0.29 | 1.00 | 0.99-1.01 | 0.98 |
| FBG per 1 mg/dL | 1.003 | 0.99-1.02 | 0.65 | 1.002 | 0.99-1.01 | 0.69 | 1.004 | 0.996-1.01 | 0.36 |
| SBP per 1mmHg | 1.01 | 0.998-1.03 | 0.09 | 1.01 | 0.99-1.02 | 0.32 | 1.01 | 0.999-1.02 | 0.08 |

IRB, initial random glucose, FBS, fasting blood sugar, SBP, systolic blood pressure

**Supplemental Table S7.** Logistic regression analysis showing the impact of multiple covariates, including raw GA, on outcomes.

|  | SHT_SITS_ | | | Any HT | | | mRS 3-6 | | |
| --- | --- | --- | --- | --- | --- | --- | --- | --- | --- |
|  | aOR | 95% CI | p-value | aOR | 95% CI | p-value | aOR | 95% CI | p-value |
| Raw GA | 1.29 | 1.11-1.50 | 0.001 | 1.25 | 1.11-1.41 | <0.001 | 1.13 | 1.03-1.25 | 0.02 |
| Age | 1.02 | 0.97-1.06 | 0.45 | 1.01 | 0.98-1.04 | 0.76 | 1.01 | 0.99-1.03 | 0.28 |
| Male | 0.87 | 0.34-2.23 | 0.78 | 0.7 | 0.35-1.40 | 0.31 | 0.72 | 0.43-1.18 | 0.19 |
| NIHSS per 1 point | 1.11 | 1.03-1.20 | 0.01 | 1.04 | 0.98-1.11 | 0.18 | 1.19 | 1.13-1.25 | <0.001 |
| TOAST |  |  |  |  |  |  |  |  |  |
| SVO |  |  |  |  |  |  |  |  |  |
| LAA | 0.85 | 0.14-5.32 | 0.87 | 1.23 | 0.35-4.29 | 0.75 | 1.51 | 0.62-3.65 | 0.36 |
| CE | 0.65 | 0.08-5.62 | 0.7 | 0.51 | 010-2.65 | 0.42 | 0.65 | 0.20-2.13 | 0.47 |
| Others | 1.14 | 0.17-7.69 | 0.9 | 0.68 | 0.17-2.72 | 0.59 | 0.97 | 0.38-2.50 | 0.94 |
| HTN | 0.75 | 0.29-1.95 | 0.55 | 0.8 | 0.40-1.61 | 0.53 | 1.18 | 0.72-1.96 | 0.51 |
| DM | 1.13 | 0.39-3.30 | 0.83 | 0.46 | 0.18-1.16 | 0.1 | 0.92 | 0.49-1.72 | 0.79 |
| A.fib | 1.34 | 0.31-5.81 | 0.69 | 2.22 | 0.65-7.60 | 0.21 | 1.45 | 0.57-3.68 | 0.44 |
| IRG per 1 mg/dL | 1.003 | 0.99-1.02 | 0.62 | 1.002 | 0.99-1.01 | 0.75 | 0.998 | 0.99-1.01 | 0.54 |
| FBG per 1 mg/dL | 1.00 | 0.99-1.01 | 0.95 | 0.999 | 0.99-1.01 | 0.85 | 1.002 | 0.99-1.01 | 0.67 |
| SBP per 1mmHg | 1.02 | 1.00-1.03 | 0.053 | 1.01 | 0.995-1.02 | 0.27 | 1.01 | 0.999-1.02 | 0.07 |

IRB, initial random glucose, FBS, fasting blood sugar, SBP, systolic blood pressure

**Supplemental Table S8.** Logistic regression analysis showing the impact of multiple variables, including GA≥16%, on outcomes in DM-only patients.

|  |  | SHT_SITS_ |  |  | Any HT |  |  | mRS 3-6 |  |
| --- | --- | --- | --- | --- | --- | --- | --- | --- | --- |
|  | aOR | 95% CI | p-value | aOR | 95% CI | p-value | aOR | 95% CI | p-value |
| GA≥16% | 4.02 | 0.12-14.49 | 0.03 | 4.48 | 1.30-15.45 | 0.02 | 2.61 | 0.92-7.45 | 0.07 |
| Age | 1.03 | 0.98-1.09 | 0.25 | 1.04 | 0.99-1.10 | 0.15 | 1.01 | 0.97-1.06 | 0.52 |
| Male | 0.80 | 0.28-2.30 | 0.68 | 0.52 | 0.18-1.50 | 0.23 | 1.12 | 0.44-2.84 | 0.81 |
| NIHSS per 1 point | 1.12 | 1.03-1.23 | 0.01 | 1.15 | 1.05-1.26 | 0.002 | 1.35 | 1.21-1.50 | <0.001 |
| TOAST |  |  |  |  |  |  |  |  |  |
| SVO |  | reference |  |  | reference |  |  | reference |  |
| LAA | 0.66 | 0.10-4.39 | 0.66 | 0.62 | 0.09-4.27 | 0.63 | 0.60 | 0.15-2.45 | 0.47 |
| CE | 0.92 | 0.15-5.77 | 0.93 | 1.38 | 0.22-8.61 | 0.73 | 0.35 | 0.08-1.50 | 0.16 |
| Others | 1.17 | 0.16-8.38 | 0.88 | 1.12 | 0.15-8.43 | 0.91 | 0.20 | 0.04-1.09 | 0.06 |
| HTN | 0.66 | 0.23-1.86 | 0.43 | 0.51 | 0.18-1.43 | 0.20 | 1.82 | 0.72-4.62 | 0.20 |
| Platelet | 1.002 | 0.99-1.01 | 0.63 | 1.002 | 0.99-1.01 | 0.65 | 1.01 | 1.001-1.01 | 0.03 |
| PT | 0.82 | 0.01-57.71 | 0.93 | 0.16 | 0.002-15.42 | 0.43 | 1.37 | 0.02-77.94 | 0.88 |

**Supplemental Table S9.** Logistic regression analysis showing the impact of multiple variables, including GA/HbA1c, on outcomes in DM-only patients.

|  | SHT_SITS_ | | | Any HT | | | mRS 3-6 | | |
| --- | --- | --- | --- | --- | --- | --- | --- | --- | --- |
|  | aOR | 95% CI | p-value | aOR | 95% CI | p-value | aOR | 95% CI | p-value |
| GA/HbA1c ratio | 3.92 | 1.28-11.99 | 0.02 | 3.75 | 1.24-11.33 | 0.02 | 2.14 | 0.76-6.03 | 0.15 |
| Age | 1.03 | 0.98-1.08 | 0.29 | 1.04 | 0.98-1.09 | 0.18 | 1.01 | 0.97-1.05 | 0.61 |
| Male | 0.92 | 0.32-2.64 | 0.88 | 0.57 | 0.20-1.58 | 0.28 | 1.20 | 0.48-3.04 | 0.70 |
| NIHSS per 1 point | 1.11 | 1.02-1.22 | 0.02 | 1.13 | 1.03-1.24 | 0.01 | 1.33 | 1.19-1.47 | <0.001 |
| TOAST |  |  |  |  |  |  |  |  |  |
| SVO |  | reference |  |  | reference |  |  | reference |  |
| LAA | 0.63 | 0.10-4.18 | 0.63 | 0.62 | 0.09-4.20 | 0.63 | 0.61 | 0.15-2.47 | 0.49 |
| CE | 0.87 | 0.14-5.58 | 0.88 | 1.39 | 0.22-8.67 | 0.73 | 0.35 | 0.08-1.51 | 0.16 |
| Others | 1.09 | 0.15-7.79 | 0.93 | 1.08 | 0.15-7.94 | 0.94 | 0.19 | 0.04-0.98 | 0.048 |
| HTN | 0.59 | 0.20-1.70 | 0.33 | 0.46 | 0.16-1.32 | 0.15 | 1.80 | 0.71-4.54 | 0.21 |
| Platelet | 1.001 | 0.99-1.01 | 0.84 | 1.001 | 0.99-1.01 | 0.87 | 1.01 | 1.00-1.01 | 0.05 |
| PT | 0.48 | 0.01-45.71 | 0.75 | 0.07 | 0.001-9.34 | 0.29 | 0.97 | 0.02-47.84 | 0.99 |

**Supplemental Table S10.** Logistic regression analysis showing the impact of multiple covariates, including the HbA1c ratio, on outcomes.

|  | SHT_SITS_ | | | Any HT | | | mRS 3-6 | | |
| --- | --- | --- | --- | --- | --- | --- | --- | --- | --- |
|  | aOR | 95% CI | p-value | aOR | 95% CI | p-value | aOR | 95% CI | p-value |
| HbA1c≥6.5% | 14.69 | 4.85-44.45 | <0.001 | 5.36 | 2.37-12.11 | <0.001 | 1.55 | 0.80-3.00 | 0.20 |
| Age | 1.02 | 0.97-1.06 | 0.51 | 1.004 | 0.97-1.04 | 0.79 | 1.01 | 0.99-1.04 | 0.22 |
| Male | 0.54 | 0.21-1.41 | 0.21 | 0.54 | 0.27-1.08 | 0.08 | 0.67 | 0.41-1.011 | 0.12 |
| NIHSS per 1 point | 1.12 | 1.03-1.22 | 0.01 | 1.04 | 0.98-1.11 | 0.17 | 1.19 | 1.13-1.25 | <0.001 |
| TOAST |  |  |  |  |  |  |  |  |  |
| SVO |  |  |  |  |  |  |  |  |  |
| LAA | 0.74 | 0.12-4.70 | 0.75 | 1.33 | 0.38-4.70 | 0.66 | 1.54 | 0.64-3.70 | 0.33 |
| CE | 0.77 | 0.09-7.03 | 0.85 | 0.59 | 0.11-3.22 | 0.54 | 0.67 | 0.20-2.19 | 0.50 |
| Others | 1.05 | 0.15-7.31 | 0.96 | 0.76 | 0.19-3.07 | 0.70 | 1.004 | 0.40-2.54 | 0.99 |
| HTN | 1.12 | 0.44-2.88 | 0.82 | 0.99 | 0.50-1.96 | 0.98 | 1.25 | 0.76-2.07 | 0.38 |
| DM | 1.24 | 0.47-3.29 | 0.66 | 0.59 | 0.26-1.35 | 0.21 | 1.11 | 0.61-2.03 | 0.73 |
| A.fib | 1.01 | 0.22-4.62 | 0.99 | 2.09 | 0.58-7.56 | 0.26 | 1.48 | 0.59-3.73 | 0.41 |
| IRG per 1 mg/dL | 1.002 | 0.99-1.01 | 0.69 | 1.001 | 0.99-1.01 | 0.92 | 0.998 | 0.99-1.01 | 0.62 |
| FBG per 1 mg/dL | 0.995 | 0.98-1.01 | 0.47 | 0.997 | 0.99-1.01 | 0.50 | 1.002 | 0.99-1.01 | 0.64 |
| SBP per 1mmHg | 1.02 | 1.003-1.04 | 0.02 | 1.01 | 0.996-1.02 | 0.19 | 1.01 | 1.00-1.02 | 0.06 |

IRB, initial random glucose, FBS, fasting blood sugar, SBP, systolic blood pressure

**Supplemental Table S11.** Baseline characteristics and functional outcome according to SHT.

|  | SHT_SITS_ (-)  n=351 | SHT_SITS_ (+)  n=27 | p value |
| --- | --- | --- | --- |
| Age (SD) | 66.8 (13.2) | 71.4 (12.7) | 0.56 |
| Male (%) | 218 (62.1) | 13 (48.1) | 0.22 |
| BMI, kg/m^2^ (SD) | 24.1 (3.3) | 22.9 (3.8) | 0.32 |
| Interval from  onset to IVT, hour (SD) | 3.0 (9.5) | 2.6 (2.6) | 0.71 |
| NIHSS, (IQR) | 9 (5-14) | 15 (11-18) | 0.001 |
| Previous stroke (%) | 64 (18.2.) | 5 (18.5) | 0.97 |
| Hypertension (%) | 175 (49.9) | 16 (59.3) | 0.43 |
| DM (%) | 82 (23.4) | 12 (44.4) | 0.02 |
| Hyperlipidemia (%) | 47 (13.4) | 5 (18.5) | 0.56 |
| Current smoking (%) | 59 (16.8) | 0 (0.0) | 0.02 |
| Atrial fibrillation (%) | 113 (32.2) | 12 (44.4) | 0.21 |
| Prior antithrombotic agents (%) | 96 (27.4) | 8 (29.6) | 0.82 |
| Stroke mechanism (%) |  |  | 0.69 |
| SVO | 42 (12.0) | 2 (7.4) |  |
| LAA | 104 (29.6) | 8 (29.6) |  |
| CE | 121 (34.5) | 12 (44.4) |  |
| Others | 84 (23.9) | 5 (18.5) |  |
| Reperfusion therapy (%) |  |  | 0.04 |
| IVT | 227 (64.7) | 12 (44.4) |  |
| Combined IVT & IAT | 124 (35.3) | 15 (55.6) |  |
| tPA dose (%) |  |  | 0.01 |
| 0.6 mg/kg | 27 (36.2) | 17 (63.0) |  |
| 0.9 mg/kg | 224 (63.8) | 10 (37.0) |  |
| Hemoglobin, mg/dL (SD) | 13.7 (2.2) | 13.3 (2.2) | 0.88 |
| LDL, mg/dL (SD) | 101.2 (37.7) | 92.6 (38.7) | 0.70 |
| Platelet count, ×1000/µL (SD) | 231.5 (81.0) | 228.3 (60.7) | 0.69 |
| Prothrombin time, INR (SD) | 1.03 (0.12) | 1.06 (0.12) | 0.40 |
| Creatinine, mg/dL (SD) | 0.98 (0.61) | 1.08 (0.58) | 0.60 |
|  |  |  |  |
| Initial random glucose, mg/dL (SD) | 133.5 (46.1) | 158.4 (42.5) | 0.96 |
| Fasting blood glucose, mg/dL (SD) | 132.4 (46.8) | 152.9 (41.3) | 0.77 |
| HbA1c, % (SD) | 6.0 (1.1) | 6.7 (0.7) | 0.08 |
| Systolic blood pressure, mmHg (SD) | 149.2 (26.2) | 1580. (32.2) | 0.70 |
| Functional outcome |  |  |  |
| mRS 3-6 | 139 (39.6) | 24 (88.9) | <0.001 |

**Supplemental Figure S1.** ROC curve showing predictive ability and cutoff point of GA and HbA1c levels for SHT in patients receiving IVT only.


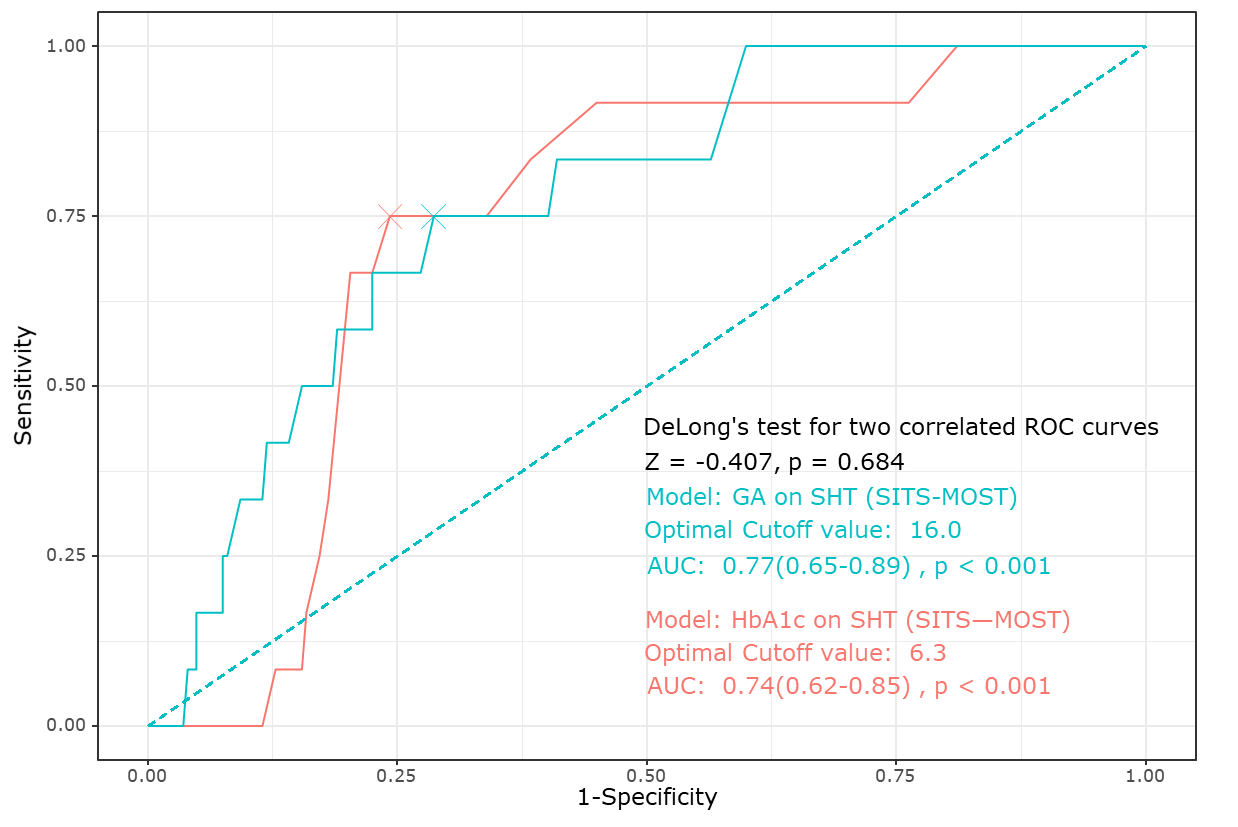

Supplement: Supplementary file 1 — Supplementary Information. [file 41598_2021_4716_MOESM1_ESM.docx]
